# *Bacillus velezensis* 83 protects *Arabidopsis thaliana* against *Botrytis cinerea* by triggering JA‐, and SA‐dependent induced systemic resistance

**DOI:** 10.1002/ps.70390

**Published:** 2025-11-19

**Authors:** Eduardo Martínez‐Terrazas, Wendy Aragón, Celia Flores Ocampo, Leobardo Serrano‐Carreón, Enrique Galindo, Mario Serrano

**Affiliations:** ^1^ Instituto de Biotecnología, Universidad Nacional Autónoma de México Cuernavaca México; ^2^ Instituto de Biociencias, Universidad Autónoma de Chiapas Tapachula México; ^3^ Centro de Ciencias Genómicas, Universidad Nacional Autónoma de México Cuernavaca México

**Keywords:** *Bacillus velezensis* 83, induced systemic resistance, *Botrytis cinerea*, jasmonic acid, salicylic acid, biological control agents

## Abstract

**BACKGROUND:**

Modern agriculture is based on the application of synthetic agrochemicals to control multiple abiotic and biotic stresses. Nevertheless, owing to their negative effect on ecosystems and human health, their application is increasingly restricted. The use of plant growth‐promoting bacteria (PGPB) as well as biological control agents (BCAs) represents a sustainable alternative to chemical pesticides and fertilizers. *Bacillus velezensis 83* (*Bv83*) has been described as a BCA against several fungal phytopathogens.

**RESULTS:**

In this work, we further study the molecular mechanisms behind the biological control capabilities of *Bv83* using the pathosystem *Arabidopsis thaliana–Botrytis cinerea*. We used an *in vitro* system that can determine whether *Bv83* can protect the plant triggering induced systemic resistance to *B. cinerea*. This protection was mediated by the accumulation of acetoin and the activation of phytohormone‐induced mechanisms, in particular, jasmonic acid‐ and salicylic acid (SA)‐mediated defense responses. Remarkably, we determined that in SA‐impaired mutants, acetoin biosynthesis was severely reduced.

**CONCLUSION:**

Our work provides valuable information that advances our understanding of the biostimulant effect induced by the BCA *Bv83*. © 2025 The Author(s). *Pest Management Science* published by John Wiley & Sons Ltd on behalf of Society of Chemical Industry.

## Graphical abstract

*B. velezensis 83* protects the plant against *B. cinerea,* accumulating acetoin and activating jasmonic acid‐ and salicylic acid‐mediated defense responses.

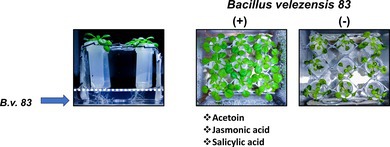

## 1 INTRODUCTION

Agriculture has the enormous challenge of covering the nutritional needs of a constantly growing human population in a sustainable manner. Plants for direct human consumption or as fodder for the animals are the main agricultural raw material, and one of the major concerns for growers is to ensure a high quality and quantity of plant‐derived products. However, crops are constantly affected by abiotic and biotic stresses that severely affect food production. To cope with biotic stresses, plants rely on local and systemic defense response mechanisms. At the local tissue site of pathogen invasion, plants recognize microbes through microbial‐ or pathogen‐associated molecular patterns (MAMPs or PAMPs). Once MAMPs or PAMPs are recognized by transmembrane pattern recognition receptors (PRRs), pattern‐triggered immunity (PTI) is induced.^1^ After PTI is triggered, plants can activate a systemic responses that are classified as systemic acquired resistance (SAR) and induced systemic resistance (ISR).^2^ Depending on the site of induction and the lifestyle of the inducing microorganism, plants can either induce SAR or ISR. Whereas SAR is induced by pathogens interacting with leaves, ISR is induced by beneficial microbes interacting with roots.^3^ Nevertheless, both systemic responses rely mostly on salicylic acid (SA)‐, jasmonic acid (JA)‐ and ethylene (ET)‐ induced pathways.^2^, ^3^

According to the Food and Agriculture Organization (http://www.fao.org/), 1.3 billion tons of food are lost every year. Around 25–50% of the production can be lost post‐harvest owing to the effect of deficient handling and storage conditions, as well as microbial diseases, particularly those caused by fungal phytopathogens.^4^ Remarkably, by avoiding the damage inflicted by these pathogens in the five most‐produced crops, >600 million people could be fed every year.^5^ To reduce the damage caused by fungal pathogens, producers routinely use synthetic fungicides to control the most harmful genera including *Alternaria, Aspergillus, Botrytis, Fusarium, Geotrichum, Gloeosporium, Penicillium, Mucor* and *Rhizopus*.^6^ Nevertheless, the excessive use of these agrochemicals has a downside, including damage to the environment and to humans, and the appearance of resistant strains, which have boosted worldwide regulatory policies and the search for ecofriendly alternatives such as biostimulants.

Biostimulants are defined as microorganisms and/or their metabolites that enhance plant growth, and abiotic and biotic stress tolerance,^7^ and are classified as microbial inoculants, humic acids, fulvic acids, protein hydrolysates and amino acids, and seaweed extracts.^8^ Modern agriculture has increased the use of microbial inoculants as biocontrol agents (BCAs) or biofertilizers, including the plant growth‐promoting rhizobacteria (PGPR).^9^ *Bacillus* species are the most widely used BCAs/PGPR, including *B. amyloliquefaciens, B. siamensis* and *B. velezensis*, which promote plant growth and protection against biotic and abiotic stresses.^10^, ^11^ Several reports have described that *Bacillus*‐induced response modify the interaction with pathogens including *Pseudomonas syringae, Agrobacterium tumefaciens, Xanthomonas campestris, Xanthomonas axonopodis, Erwinia amylovora, Botrytis cinerea, Fusarium oxysporum, Colletotrichum gloeosporioides, Rhizoctonia solani* and *Penicillium expansum*.^12^ Indeed, we have developed a biofungicide based on *Bacillus velezensis* 83 (*Bv83*) marketed as Fungifree AB™. Application of Fungifree AB™ was shown to protect mango, avocado, papaya, citrus, tomato, strawberry, blueberry, blackberry and cucurbits plants against the fungal pathogens *Colletotrichum, Erysiphe, Botrytis, Sphaerotheca* and *Leveillula*.^13^ *B. velezensis* has been shown to directly or indirectly interact with multiple plants species to promote biological control by competing for space and nutrients with the pathogens and/or triggering the ISR.^14^, ^15^ Additionally, *Bacillus* strains act as biostimulant through promoting plant growth by phytohormones (auxins), enzymes (ACC (1‐aminocyclopropane‐1‐carboxylate) deaminase) or volatile organic compounds (VOCs) such as acetoin.^16^, ^17^, ^18^, ^19^

As a consequence of this complex matrix of *Bacillus*‐induced responses, the molecular changes that take place in the microorganism and/or in the plants have only partly been characterized. For this reason, the development of methodologies to characterize their potential mode‐of‐action (MoA) are needed.^20^ In this study, we report an *in vitro* hydroponic system that allowed us to characterize the molecules produced by the bacteria as well as transcriptomic plant defense responses triggered by the direct or indirect interaction between *Bv83* and the plant model *A. thaliana*. We determined that *Bv83* protects the plant against the necrotrophic fungus *B. cinerea* by triggering ISR. Additionally, we observed that the protection was mediated by the accumulation of acetoin and the activation of JA‐ and SA‐mediated defense responses. Finally, we determined that production of acetoin is mediated by the synthesis of the phytohormone SA. This information can be used to further understand and improve the biostimulant action of *Bv83*.

## 2 MATERIALS AND METHODS

### 2.1 Plant material and growth conditions

**Figure 1:**
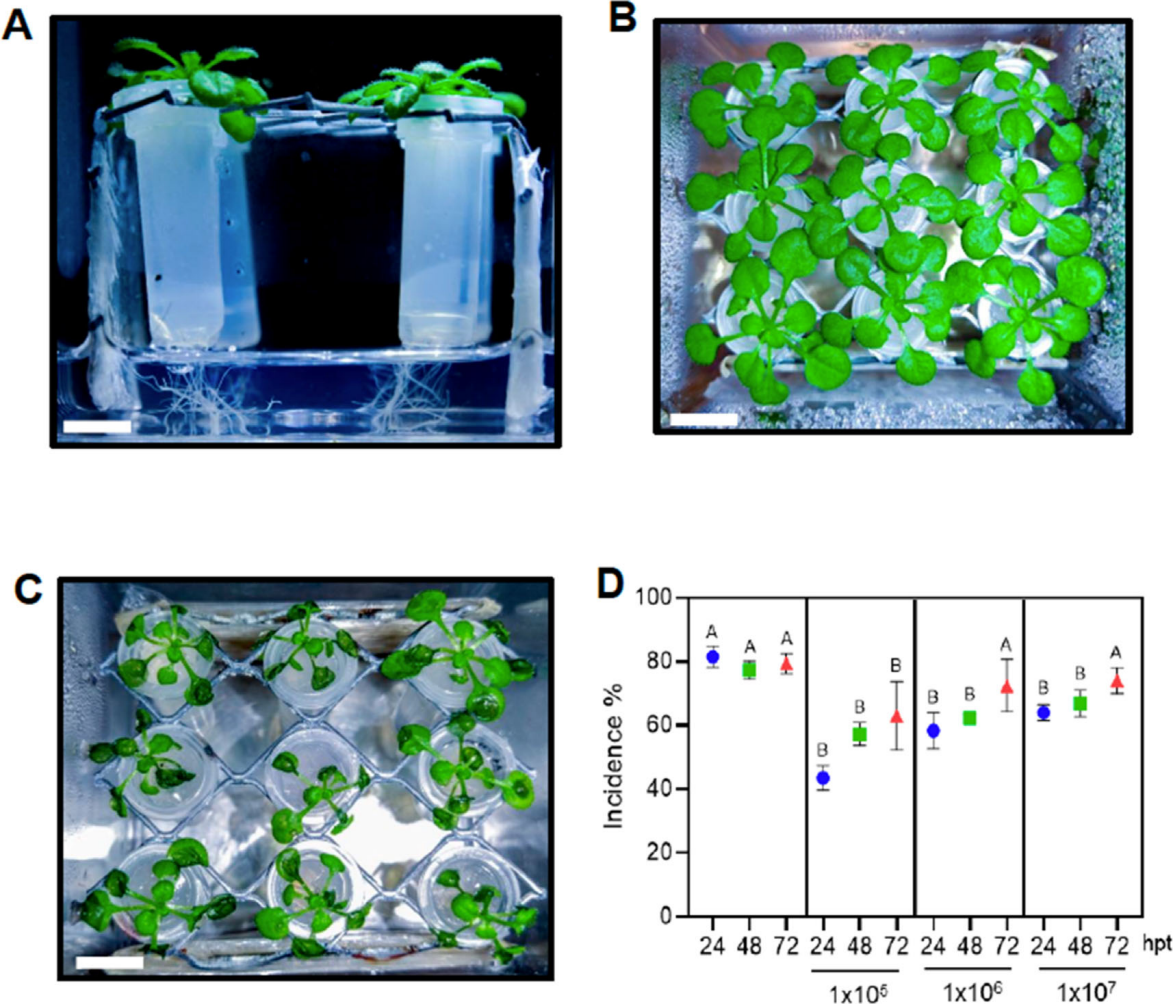
*Bacillus velezensis 83* protects *A. thaliana* against *B. cinerea*. (A) *In vitro* growth system consisted of an Eppendorf microtube filled with solid MS media and with a hole in the bottom. Microtubes containing the seedlings were placed into a magenta box (77 mm × 77 mm × 97 mm) containing 20 mL MS liquid media, avoiding the direct contact with the microtubes. (B) Representative picture of *A. thaliana* seedlings 15 days postgermination grown under 12 h:12 h, light:dark photoperiod. (C) Representative picture of *A. thaliana* plants infected with *B. cinerea* at 72 hpi. (D) *A. thaliana* roots were treated with a bacterial concentration of 1 × 10^5^, 1 × 10^6^ and 1 × 10^7^ CFU mL^−1^ into the sterile MS liquid media 24, 48 or 72 hpt. Afterwards, 5 μL of 5 × 10^4^ spores mL–1 of *B. cinerea* were placed on each leaf and incubated for 72 h. The disease incidence was expressed by the percentage of plants showing disease symptoms extending beyond the inoculation site. Histograms represent the mean values (± SE) of three independent experiments. Letters indicate a statistically significant difference, according to one‐way ANOVA (*P* ≤ 0.001) followed by a Tukey's HSD test. Bars = 1 cm.

*Arabidopsis thaliana* ecotype Columbia‐0 (Col‐0) was obtained from the Nottingham *Arabidopsis* Stock Centre (Nottingham, UK). The following *A. thaliana* mutants (all in the Col‐0 background) were used and previously described: *jar1‐1*,^21^ *ein3‐1* ^22^ and *npr1‐1*.^23^ Plants grown under *in vitro* conditions were generated from surface‐sterilized seeds germinated on square Petri dishes containing Murashige & Skoog (MS) media supplemented with 0.8% agar and 1% sucrose. Plates were kept at 4 °C for 2 days and afterwards incubated at 21 °C for 4 days in a growth chamber with a 12 h:12 h, light:dark photoperiod. Seedlings were then transplanted to a special system that allows the plants to grow in two, separated chambers in which the roots have access to a culture media and the leaves have no contact with possible volatiles produced by the beneficial bacteria. Metabolites produced by the roots and or the bacteria can be measured in the liquid that is in contact with the roots. The top of the system consisted of an Eppendorf microtube filled with solid MS media and with a hole in the bottom. Microtubes containing the seedlings were placed into a magenta box (77 mm × 77 mm × 97 mm) containing 20 mL sterile MS media, avoiding direct contact with the microtubes. Magenta boxes were then incubated for 15 days in a growth chamber with a 12 h:12 h, light:dark photoperiod. After this time, plant roots reached the bottom of the microtube and entered in contact with the liquid media [Fig. 1(A), (B)].

### 2.2 B. velezensis 83 and B. cinerea inoculation

*Bacillus velezensis* 83, from the Belgian Coordinated Collection of Microorganisms (BCCM, accession no. LMG S‐30921) was obtained from the powdered commercial formulation Fungifree AB™ (Agro&Biotecnia S. de R.L. de C.V.). *Bv83* was grown in 500‐mL shake flasks containing 50 mL Luria–Bertani (LB) medium and incubated at 30 °C and 200 rpm for 12 h. Fifteen mL of culture was centrifuged for 10 min at 6500 × *g*, the pellet washed three times in 0.85% saline solution and resuspended in 10 mL distilled water. To inoculate *A. thaliana* roots, bacterial concentration was adjusted to 1 × 10^5^, 1 × 10^6^ and 1 × 10^7^ colony‐forming units (CFU) mL^−1^ and inoculated into the sterile MS liquid media. *Botrytis cinerea* strain B05.10 was provided by Brigitte Mauch‐Mani (University of Neuchatel, Switzerland). Growth, preparation of spore suspension and infection procedure were performed as described previously.^24^ Five μL of 5 × 10^4^ spores mL^−1^ diluted in potato dextrose broth (PDB) were placed over each leaf and incubated for 72 h [Fig. 1(C)]. The disease incidence was expressed by the percentage of plants showing disease symptoms extending beyond the inoculation site.

### 2.3 Induction of systemic induced resistance (ISR)

In order to determine if the ISR induced by *Bv83* was related to its 2,3‐butanediol and acetoin production. These compounds were applied individually or mixed directly with the liquid media in the Magenta box, to final concentrations of 200 mg L^−1^. Microtubes containing the seedlings of *A. thaliana* were sealed at the bottom with plastic film, to avoid the roots from encountering *Bv83* or with the compounds to be evaluated, and at 24 h post‐treatment (hpt) *A. thaliana* leaves were infected with *B. cinerea*, and the plants were incubated for 72 h in a growth chamber.

### 2.4 Quantitative RT‐PCR analysis

Total tissue from leaves of wild‐type (WT) plants inoculated and noninoculated with *Bv83* and infected and noninfected with *B. cinerea* [6 and 48 h postinoculation (hpi)] were collected directly into liquid nitrogen and stored at −80 °C. Fresh tissue (100 mg) was used for RNA isolation from a pool of leaves from six plants for each treatment using Plant/Fungi Total RNA Purification (Norgen Biotek Corp., Thorold, Canada), following the manufacturer's instructions. Sample quality was assessed using a NanoDrop 1000 Spectrophotometer (Thermo Fisher Scientific, Inc., Waltham, MA, USA). A 1‐μg sample of total RNA was treated with Turbo‐DNAse (Ambion Inc./Thermo Fisher scientific) and used as the template for cDNA synthesis with a RevertAid First Strand cDNA Synthesis Kit (Thermo Fisher Scientific). Quantitative reverse transcription (qRT)‐PCR reactions contained cDNA (diluted 1/40) in Maxima SYBR Green/ROX qPCR Master Mix (2×) (Thermo Fisher Scientific) and 0.5 mm of specific primers. Primers for the qRT‐PCR gene expression have been described previously:^25^, ^26^ *AOS* (AT5G42650) AOS‐fw 5'‐GTGGATTCTCGGCGATAAAA‐3′ and AOS‐rev 5'‐ATCCAAAGATCTCCCGATCC‐3′; *ACS6* (AT4G11280) ACS6‐fw 5'‐TGGTTGGTTAAAGGCCAAAG‐3′ and ACS6‐fw 5'‐TGGTCCATATTCGCAAAACA‐3′; and *NPR1* (AT1G64280) NPR1‐fw 5′‐ TCTTGCCGATGACAACCAGC‐3′ and NPR1‐fw 5′‐ GCCTTTGAGAGAATGCTTTA‐3′. All of the reactions were performed in 96‐well plates using the 7300 Real‐Time PCR System and 7300 System software (Applied Biosystems, Foster City, CA, USA). Three independent experiments were analyzed, each with three technical replicates. qRT‐PCR conditions were performed as described previously.^24^ Normalized gene expression was determined using the comparative method previously described^27^. The actin (AT3G18780), and CF150 (AT1G72150) genes were used as internal controls and normalization genes.^28^, ^29^

### 2.5 Acetoin and 2,3‐butanediol quantification

Acetoin and butanediol were analyzed by reverse phase high‐performance liquid chromatography (HPLC) using methodology reported previously.^30^ Briefly, 20 μL of the sample were loaded to an Aminex HPX‐87H column (7.8 × 300 mm; Bio‐Rad Laboratories Inc., Hercules, CA, USA) and separated by using a 2695 HPLC system (Waters Corp., Milford, MA, USA). Acetoin was determined by absorbance at 210 nm and butanediol using a refractive index detector.

### 2.6 Statistical analysis

One‐way ANOVA, followed by Tukey's honestly significant difference (HSD) test, was performed to determine statistical significance using the software minitab 19 (https://www.minitab.com/). All graphs were generated using the software prism8 v8.0.2 (GraphPad Software, San Diego CA, USA). Data represent the mean ± SE. Differences at *P* < 0.001 were considered significant.

## 3 RESULTS

### 3.1 B. velezensis 83 protects A. thaliana against B. cinerea

*Bacillus velezensis* has been shown to trigger ISR,^14^, ^15^ yet the molecular mechanisms behind this protection are not fully understood. To study *Bv83* ‐induced ISR under controlled conditions, we developed an *in vitro* system that allows the roots to be grown while avoiding contact (if desired) with the bacteria inoculated into the liquid media. In this way, we can study the plant systemic effect induced by the direct and indirect contact with *Bv83* [Fig. 1(A)]. At 4 days postgermination *A. thaliana* seedlings were placed on the top of Eppendorf microtube filled with MS media. Microtubes with a hole in the bottom, were placed into a magenta box containing sterile water without touching the microtubes. At 15 days postgermination, roots reached the bottom of the microtube and entered in contact with the liquid media [Fig. 1(A)]. With this system, we have grown full‐sized plants [Fig. 1(B)] and effectively infected them with *B. cinerea*, because we observed the typical water‐soaking symptoms on *A. thaliana* leaves [Fig. 1(C)].

Different concentrations of *Bv83* (1 × 10^5^, 1 × 10^6^ and 1 × 10^7^ CFU mL^−1^) were added to the liquid media and roots were incubated for 24, 48 and 72 hpt. After this time, one droplet of PDB containing *B. cinerea* spores (5 × 10^4^ spores mL^−1^) was applied on each leaf and incubated for 72 hpi, after which the disease incidence was measured [Fig. 1(D)]. The lowest bacterial dilution (1 × 10^5^) showed a statistically significant reduced incidence at all time points analyzed, compared to untreated control plants. The protective effect against *B. cinerea* was observed at 24 and 48 hpt using 1 × 10^6^ and 1 × 10^7^ cells [Fig. 1(D)]. These results indicate that ISR can be reached using the lowest number of bacterial cells and that our *in vitro* system can be used to characterize *Bv83*‐dependent ISR against *B. cinerea*. But even more importantly, the growth system allowed us to analyze, in a controlled environment, the different metabolites produced by the bacteria and by the plant in the media or as volatile compounds, and their effect over the plant pathogen.

### 3.2 B. cinerea stimulates the biosynthesis of acetoin produced by B. velezensis 83

**Figure 2:**
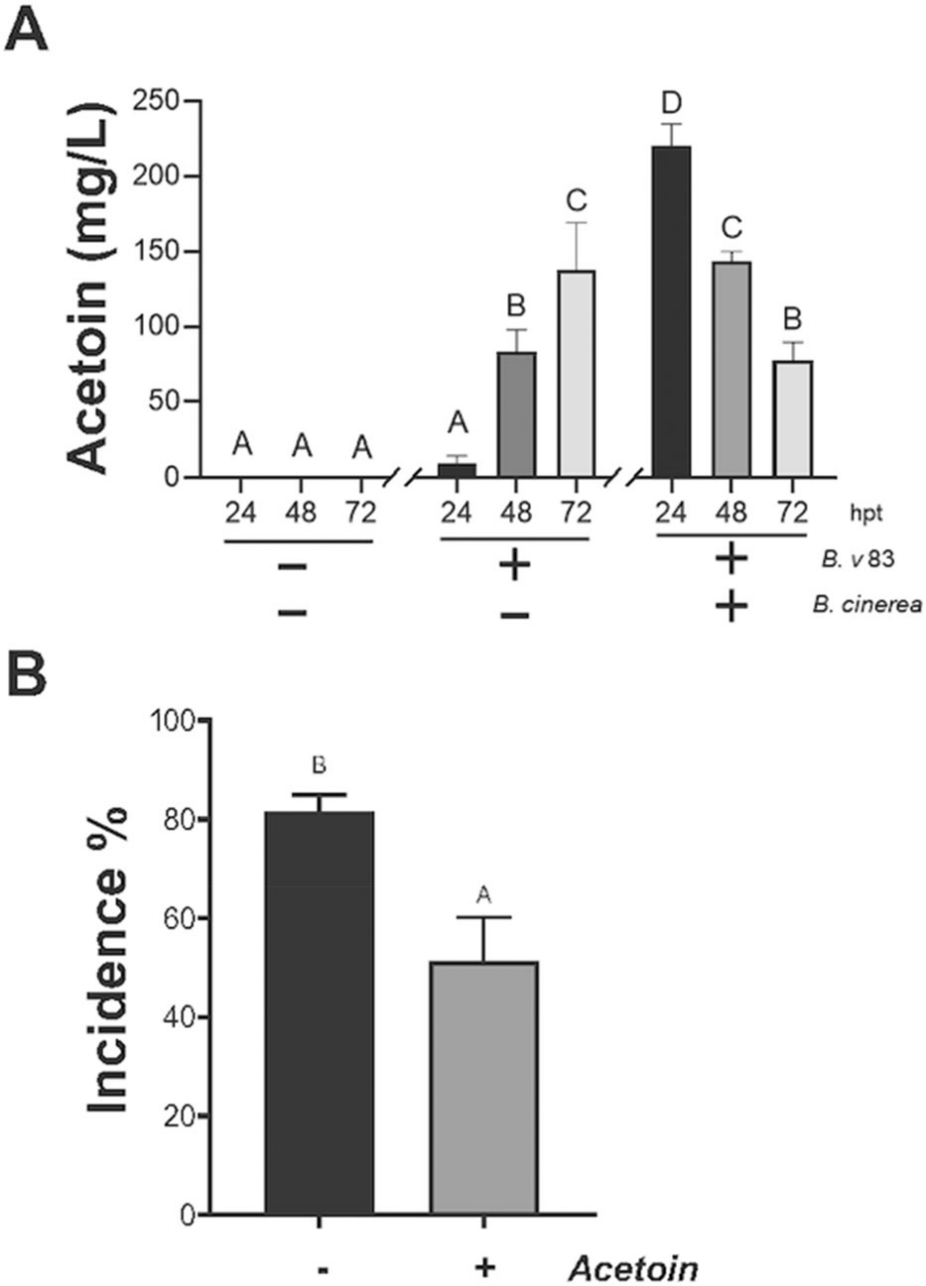
*Botrytis cinerea* stimulates the biosynthesis of acetoin produced by *B. velezensis 83*. (A) Acetoin present in the liquid media was quantified at 24, 48 and 72 hpt on the presence of *Bv*83 and with the plant infected by *B. cinerea* in the presence of the bacteria. (B) *A. thaliana* roots were incubated with acetoin (100 mg L^−1^) and at 24 hpt, 5 μL of 5 × 10^4^ spores mL^−1^ of *B. cinerea* were placed on each leaf and incubated for 72 h. The disease incidence was expressed by the percentage of plants showing disease symptoms extending beyond the inoculation site. Histograms represent the mean values (± SE) of three independent experiments. Letters indicate a statistically significant difference, according to one‐way ANOVA (*P* ≤ 0.001) followed by a Tukey's HSD test.

Plant growth‐promoting rhizobacteria, such as *B. velezensis*, can produce 2,3‐butanediol (2,3‐BD) and its precursor 3‐hydroxy‐2‐butanone (acetoin), which act as elicitors of ISR.^31^, ^32^, ^33^, ^34^ To characterize the possible role of acetoin during the defense response under our *in vitro* conditions, we determined if acetoin was present in the liquid media in the presence of the bacteria alone and if the plant was infected by the fungus [Fig. 2(A)]. At 24 hpt we detected basal levels of acetoin in the media containing *Bv83* alone and this level increased over the time, reaching ≈140 mg L^−1^ at 72 hpt. In the presence of the pathogen, ≈200 mg L^−1^ was found at 24 hpt and this concentration decreased to 80 mg L^−1^ by 72 hpt [Fig. 2(A)]. We then verified that exogenous application of acetoin (100 mg L^−1^) led to ISR against *B. cinerea* in *A. thaliana* [Fig. 2(B)]. This result suggests that at soon after inoculation, bacterial cells can trigger the biosynthesis of this metabolite, further stimulating ISR in the plant.

Volatile organic compounds, including 2,3‐butanediol and their precursor acetoin, have been described to inhibit the effect of various plant pathogens.^31^, ^34^ To determine if the ISR, induced by *Bv83* is caused by the effect of the volatile nature of the 2,3‐butanediol or acetoin we applied the bacteria or 200 mg L^−1^ of 2,3‐butanediol and/or acetoin directly to the liquid media, avoiding the direct contact with the plant roots. At 24 hpt we performed the infection with *B. cinerea*. A similar disease incidence was found in the presence of all the metabolites alone or when mixed with the bacteria, as well as in the nontreated plants (Supporting Information, Fig. S1). This suggests that the direct contact of the roots either with the bacteria or with the metabolite is necessary to trigger an effective ISR.

### 3.3 B. velezensis 83 transcriptionally activates JA‐, ET‐ and SA‐signaling pathways

**Figure 3:**
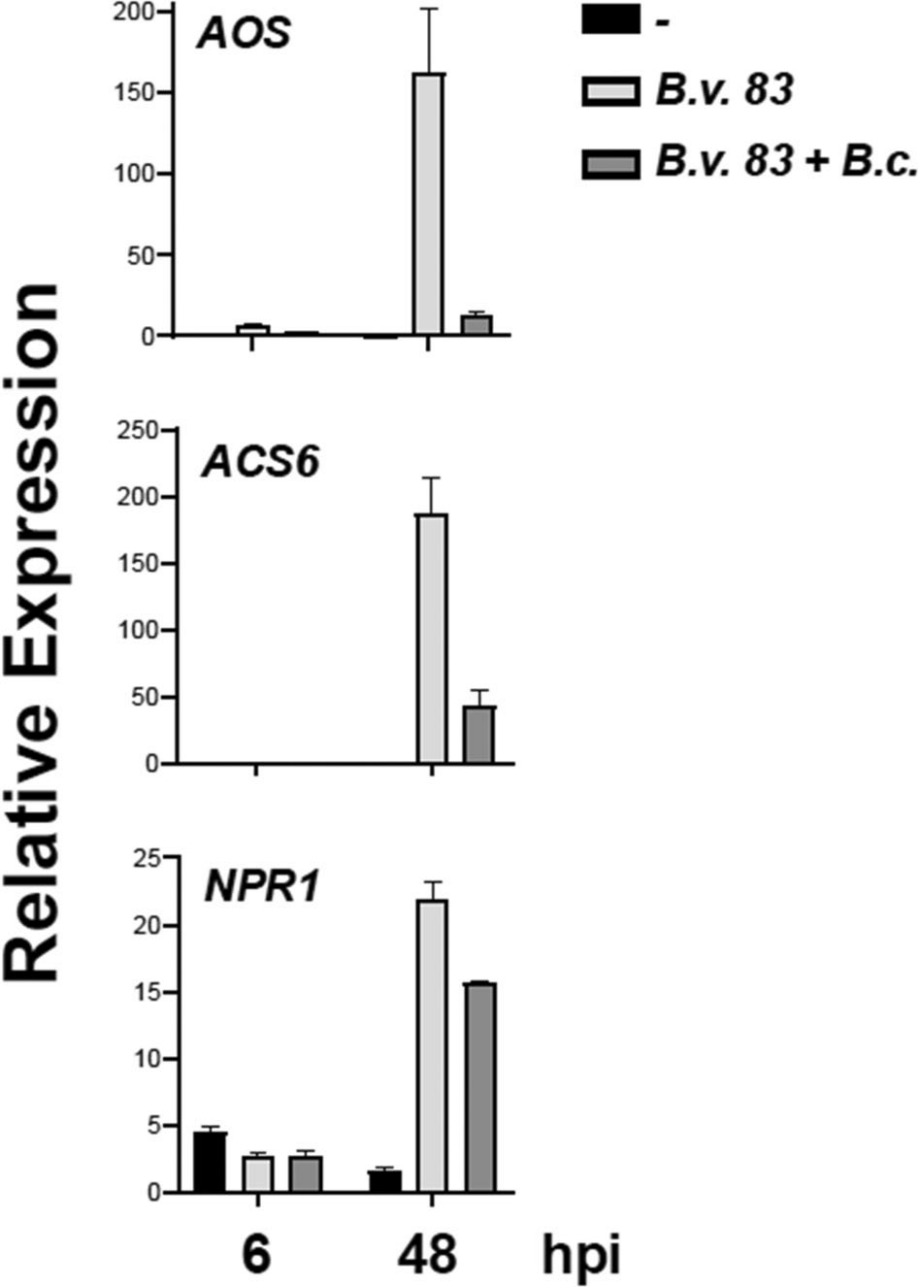
*Bacillus velezensis 83* induced *JA‐, ET‐* and *SA‐*signaling pathways. Transcriptional activation of marker genes *AOS*, *ACS6* and *NPR1* from the JA‐, ET‐ and SA‐induced pathways, respectively, was analyzed by qRT‐PCR of total RNA extracted from *A. thaliana* plants incubated with *Bv83* (at 24 hpt) and/or infected with *B. cinerea* (at 6 and 48 hpi). Three independent experiments were analyzed, each with three technical replicates. Relative gene expression level for each sample was calculated using the comparative Ct method and normalized with the geometrical mean of two housekeeping genes *CF150* and *ACT2*.

Induced systemic resistance is triggered when nonpathogenic rhizobacteria activate SA‐, JA‐ and/or ET‐induced pathways.^35^, ^36^, ^37^, ^38^ Total RNA from plants incubated with *Bv83* (24 hpt) and/or infected with *B. cinerea* (6 and 48 hpi) was isolated to characterize the transcriptional activation of previously described marker genes *AOS*, *ACS6* and *NPR1* from the JA‐, ET‐ and SA‐induced pathways, respectively (Fig. 3). In both treatments, transcripts of JA‐ and ET‐induced genes were accumulated when compared to nontreated plants 48 hpi. However, for both JA‐ and ET‐induced genes (*AOS* and *ACS6*), at 6 hpi we observed strong induction when plants were treated only with the bacteria, whereas a transcriptional inhibition was quantified when plants were further infected with *B. cinerea* (Fig. 3). At 6 hpi the expression of SA‐induced gene *NPR1* was moderated reduced during the interaction with the bacteria and infected with the fungus, compared to control plants, whereas at 48 hpi *NPR1* show similar expression patterns as JA‐ and ET‐induced genes (Fig. 3). It should be pointed out that *NPR1* expression 48 hpi during bacteria and pathogen interaction was similar to that obtained with the plants inoculated only with *Bv83*, whereas expression of *AOS* and *ACS6* genes was severely reduced in such conditions. These results agree with the response induced during ISR, because SA‐, JA‐ and ET‐induced genes are induced after that interaction with *Bv83*.

### 3.4 B. velezensis 83‐induced defense responses are dependent on JA‐, ET‐ and SA‐signaling pathways

**Figure 4:**
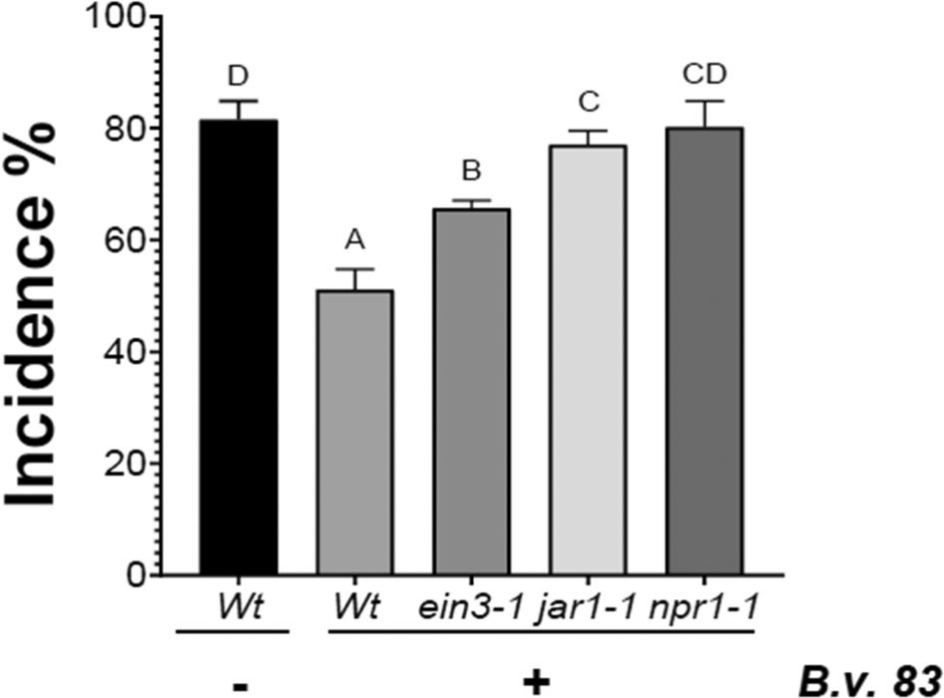
*Bacillus velezensis 83‐*induced defense responses are dependent of JA, ET and SA. Roots of *A. thaliana* mutants *ein3‐1*, *jar1‐1* and *npr1‐1* and Col‐0 WT plants were incubated with *B. velezensis* (at 24 hpt) and afterwards infected with *B. cinerea*. The disease incidence was expressed by the percentage of plants showing disease symptoms (72 hpi) extending beyond the inoculation site. Histograms represent the mean values (± SE) of three independent experiments. Letters indicate a statistically significant difference, according to one‐way ANOVA (*P* ≤ 0.001) followed by a Tukey's HSD test.

Protection induced by ISR against biotic stresses, including the attack by *B. cinerea*, is dependent on JA‐, ET‐ and SA‐signaling pathways.^36^, ^37^ To determine if these phytohormone‐induced mechanisms are necessary to reach *Bv83*‐tiggered defense response, we incubated the ET‐, JA‐ and SA‐insensitive mutants, *ein3‐1*, *jar1‐1* and *npr1‐1*, respectively, with the bacteria and afterwards infected them with *B. cinerea* (Fig. 4). Compared to WT plants, all mutants showed a reduced protection conferred by the bacteria to different degrees. *ein3‐1* showed an increase of 20% incidence compared to WT‐treated plants, whereas *jar1‐1* and *npr1‐1* presented similar incidence as WT plants without *Bv83* (Fig. 4). This result suggests the participation of all three phytohormone signaling pathways, yet JA‐ and SA‐dependent responses might have a bigger impact during the ISR triggered by *Bv83* against *B. cinerea*.

### 3.5 Exogenous application of acetoin cannot bypass phytohormone‐dependent ISR inhibition

**Figure 5:**
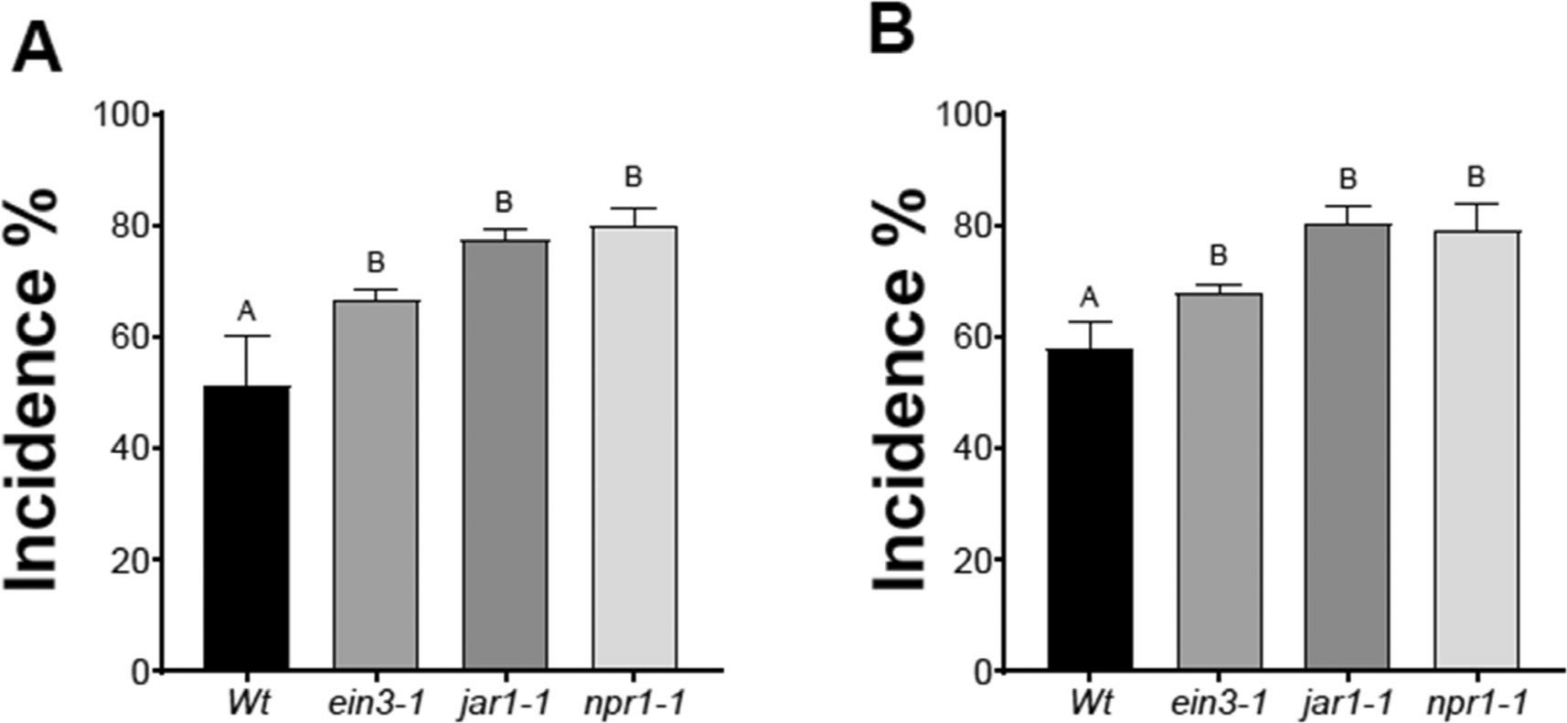
Acetoin‐induced defense response is dependent of ET‐, JA‐ and SA‐signaling pathways. Roots of *A. thaliana* mutants *ein3‐1*, *jar1‐1* and *npr1‐1* and Col‐0 WT plants were incubated with 100 and 200 mg L^−1^ acetoin (A and B, respectively) and afterwards infected with *B. cinerea*. The disease incidence was expressed by the percentage of plants showing disease symptoms (72 hpi) extending beyond the inoculation site. Histograms represent the mean values (± SE) of three independent experiments. Letters indicate a statistically significant difference, according to one‐way ANOVA (*P* ≤ 0.001) followed by a Tukey's HSD test.

We determined that exogenous application of acetoin can protect *A. thaliana* plants against *B. cinerea* (Fig. 2) and that *Bv83*‐induced defense responses are dependent on JA‐, ET‐ and SA‐signaling pathways (Fig. 4). Next, we investigated whether acetoin‐induced protection also is dependent on these phytohormone‐induced defenses. Wild‐type plants and ET‐, JA‐ and SA‐insensitive mutants were incubated for 24 h with 100 and 200 mg mL^−1^ acetoin [Fig. 5(A), (B), respectively] and infected with *B. cinerea*. The WT plants showed an incidence of 50% and 55% when were treated with 100 or 200 mg mL^−1^, respectively. For all mutants the incidence was in a range of 60% for *ein3‐1* to 80% for *jar1‐1* and *npr1‐1* (Fig. 5). This indicates that the acetoin‐induced defense response also is dependent of ET‐, JA‐ and SA‐signaling pathways.

### 3.6 Acetoin biosynthesis is modified by JA‐ and SA‐signaling pathways

**Figure 6:**
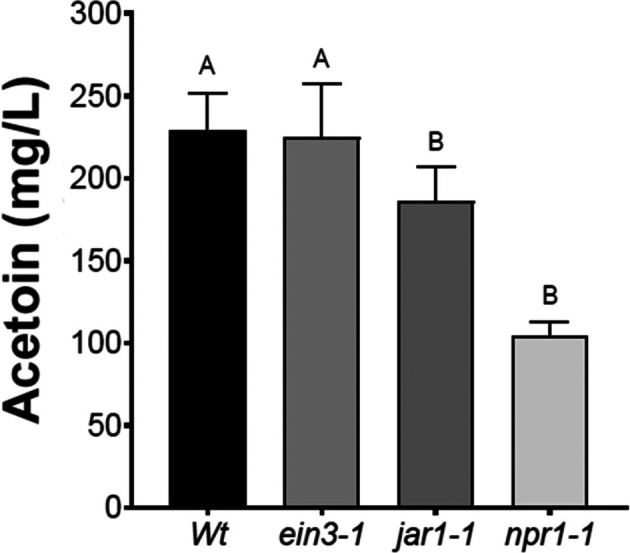
Acetoin biosynthesis is modified by JA‐ and SA‐signaling pathways. *A. thaliana* mutants *ein3‐1*, *jar1‐1* and *npr1‐1* and Col‐0 WT plants were incubated with *Bv83* and at 24 hpt acetoin in the liquid media was quantified. Histograms represent the mean values (± SE) of three independent experiments. Letters indicate a statistically significant difference, according to one‐way ANOVA (*P* ≤ 0.001) followed by a Tukey's HSD test.

Plant root exudates have been described as improving growth and induction of ISR mediated by *B. cereus*.^39^ Additionally, root exudates from SA‐related mutants modified PGPR growth and development.^40^ With this in mind, we asked whether inhibition of ET‐, JA‐ and SA‐induced responses can modify acetoin biosynthesis in *Bv83*. Mutant plants of each phytohormone signaling pathway were incubated with *Bv83* and at 24 hpt acetoin in the liquid media was quantified (Fig. 6). ET‐insensitive mutant *ein3‐1* accumulated similar levels of acetoin as the WT, whereas *jar1‐1* and *npr1‐1* shown a reduction to 180 and 100 mg L^−1^ acetoin, respectively, compared to ≈220 mg L^−1^ accumulated in the WT plant (Fig. 6).

Based on the strong acetoin reduction observed with *npr1‐1*, we studied if exogenous application of SA can modify acetoin and butanediol biosynthesis in *Bv83*. Increasing concentrations from 50 to 400 mg L^−1^ SA did not modify the concentration of these metabolites exudated by *Bv*83 (Table 1). This leaves unanswered the question of how the mutation in *NPR1* leads to a strong reduction of acetoin (Fig. 6).

**Table 1:** Acetoin and 2,3–butanediol production by *Bv83* as a function of SA concentration in culture medium

Treatments	Acetoin + 2,3‐butanediol (g L^−1^)		
	36 h cultivation	42 h cultivation	48 h cultivation
Control	1.96^a^	4.7^a^	4.5^a^
Control + ethanol	1.3^b^	4.4^a^	4.9^a^
SA 50 mg L^−1^	1.48^b^	4.9^a^	5^a^
SA 100 mg L^−1^	0.87^c^	4.9^a^	4.7^a^
SA 200 mg L^−1^	0	1.71^b^	3.6^b^
SA 400 mg L^−1^	0	0	0

Letters indicate a statistically significant difference (Tukey's HSD, *n* = 3, *P* ≤ 0.05).

## 4 DISCUSSION

The results presented in this study revealed a complex and interconnected series of mechanisms through which *Bv83* induces ISR in *A. thaliana* against *B. cinerea*. The key findings and their implications are discussed below:

### 4.1 Efficacy of B. velezensis 83 in inducing ISR

The study demonstrated that *Bv83* induced ISR in *A. thaliana*, significantly reducing the incidence of *B. cinerea* infection. It was shown that the lowest bacterial cell concentration (1 × 10^5^) was sufficient to achieve significant protection at all time points analyzed, whereas higher concentrations were only effective at early stages (24 and 48 hpt). Our results are in agreement with a study conducted by Balderas‐Ruíz *et al*.,^41^ using cells of the same strain, which decreased the incidence of infection caused by *B. cinerea* in tomato leaves and fruits. This finding highlights the efficiency of *Bv83* in inducing ISR with a minimal number of bacterial cells, which is crucial for its potential application in agricultural settings.

### 4.2 Role of acetoin in ISR

Many *Bacillus* strains can produce acetoin (3‐hydroxy‐2‐butanone) as a VOC that induces ISR in *Arabidopsis*. Our results showed an increased production of acetoin by *Bv83* in the presence of the pathogen *B. cinerea*. Although VOCs such as 2,3‐butanediol and its precursor acetoin applied alone have been described to inhibit the effect of various plant pathogens,^40^, ^41^ this study found that direct contact with the plant roots, either with the bacteria or with the metabolite, is necessary to trigger an effective ISR. This was evident as similar infection incidences were recorded in the presence of all metabolites alone (or mixed), as well as with the bacteria, comparable to nontreated plants. This suggests that VOCs alone may not be sufficient to induce ISR without direct interaction with plant roots.

Notably, this conclusion contrasts with previous reports suggesting that VOCs such as acetoin and 2,3‐butanediol are sufficient to trigger ISR. A more detailed analysis is needed to reconcile this discrepancy. Potential factors could include differences in the experimental growth systems, concentrations of VOCs used, plant developmental stages, or specific plant–pathogen interaction contexts. Besides that, the inducible plant responses triggered by fungal pathogenesis include the induction of root secretions that effectively recruit *Bv83* in the rhizosphere, through secondary metabolites such as bacterial quorum sensing (QS) molecules, further enhancing ISR responses in the plant.^3^ This discovery underscores the importance of acetoin as a crucial mediator in the defense response induced by *Bv83*.

### 4.3 Transcriptional activation of JA‐, ET‐, and SA‐signaling pathways

The study found that *Bv83* transcriptionally activates genes associated with JA, ET and SA signaling pathways in *Arabidopsis*. *Bacillus* spp. as biological control agent can stimulate plant systemic resistance, including the signalization of the pathway of phytohormones, which are essential for regulating plant defense responses.^36^, ^37^ The transcripts of JA‐ and ET‐induced genes (*AOS* and *ACS6*, respectively) accumulated significantly compared to nontreated plants, with a stronger induction observed when plants were treated with the bacteria alone. Conversely, the SA‐induced gene *NPR1* showed a moderate reduction in expression during early infection stages, but similar expression patterns to JA‐ and ET‐induced genes at later stages. These results align with the expected ISR response, where multiple phytohormone signaling pathways are involved.

### 4.4 Phytohormone‐dependent ISR responses

The study also examined the necessity of JA‐, ET‐ and SA‐signaling pathways in *Bv83*3‐triggered ISR. Mutants insensitive to these phytohormones (*ein3‐1*, *jar1‐1* and *npr1‐1*) showed varying degrees of reduced protection compared to WT plants. This indicates that all three signaling pathways are involved in the ISR response, with JA‐ and SA‐dependent responses having a more significant impact. The plant hormones SA, JA and ET are essential for regulating plant defense responses. These responses often converge on different phytohormone pathways that interact with each other to optimize fitness.^42^ In *Arabidopsis* mutants deficient in JA, SA and ET signaling, the beneficial microbial‐induced ISR in plants is largely compromised.^15^, ^36^, ^43^, ^44^

### 4.5 Exogenous application of acetoin

Studies of *Bacillus*‐derived acetoin‐mediated effects on plants have focused on how this influences the signaling of phytohormones such as SA, JA and ET.^19^, ^45^, ^46^, ^47^ Our results showed that exogenous application of acetoin induce protection against *B. cinerea* in WT plants. However, this protection also was dependent on JA‐ and SA‐signaling pathways, as evidenced by the reduced protection in the corresponding *Arabidopsis* mutants *jar1‐1* and *npr1*, respectively. Similar results were obtained with exogenous application of acetoin to triggered ISR against *Pseudomonas syringae* pv. tomato DC3000 in *Arabidopsis*.^45^, ^47^ This suggests that acetoin‐induced defense response is not independent of these phytohormone pathways.

### 4.6 Impact of phytohormone signaling on acetoin biosynthesis

Components of the phytohormone signaling pathway influence acetoin biosynthesis, such as NPR1, SA‐ and ET‐induced resistance against *P. syringae* pv. *tomato* DC3000 infections.^45^ The authors showed that treating *A. thaliana* with acetoin failed to protect SA‐deficient *NahG* and ethylene mutant *etr1‐3* plants from DC3000 infection. Moreover, results with *B. subtilis* FB17 treatment indicated that the function of *B. subtilis*‐derived acetoin operates through an NPR1‐dependent pathway and requires SA and ET components, but is independent of JA, to induce resistance against DC3000 infections. They also evaluated the role of acetoin in the induction of ISR using *B. subtilis* strains impaired in acetoin production. Greater *P. syringae* symptom development was observed in plants treated with acetoin biosynthetic mutants compared to plants treated with WT strains.^45^

Our study explored the effect of phytohormone signaling on acetoin biosynthesis in *Bv83*. Whereas the ET‐insensitive mutant (*ein3‐1*) accumulated similar levels of acetoin as the WT, the JA‐ and SA‐insensitive mutants (*jar1‐1* and *npr1‐1*) showed reduced acetoin levels. Interestingly, acetoin production decreased in *npr1* mutants but could not be restored by exogenous SA, and exogenous SA application did not alter acetoin or butanediol biosynthesis in general. This unresolved contradiction suggests that NPR1 regulation of acetoin production may occur through SA‐independent mechanisms, such as cross‐talk with other signaling pathways. Further characterization of *npr1‐1* mutants tested of a profile of compounds of root exudates, alone and in interaction with *Bv83*, focusing in biofilm formation will be crucial to clarify the molecular mechanisms underlying pathogen‐induced SA accumulation and SA‐mediated defense signaling in plants before and after acetoin application. Moreover, it is tempting to hypothesize that root‐derived metabolites such as simple sugars (glucose and fructose) and organic acids (malic acid, oxalic acid or citric acid) act as signaling molecules and can activate the *alsSD* operon in *B. subtilis*, thereby providing a direct mechanistic link between plant signaling and microbial acetoin biosynthesis.

### 4.7 Advantages of using the *in vitro* hydroponic system for biocontrol analysis

Owing to the plethora of microorganisms coexisting with the plants and the complex environmental conditions such as soil composition, the molecular events taking place during the plant–microbe interactions are difficult to study under natural circumstances. To avoid these limitations, for decades *in vitro* systems have been used to grow the plants under controlled environments, including hydroponic systems. Some of the advantages using hydroponics are the easy accessibility to plant tissues, manipulation of nutrients contained in the growth medium and the possibility to generate fully grown plants.^48^

One of the significant advantages of using the *in vitro* hydroponic system for analyzing biocontrol mechanisms is the precise control it provides over environmental conditions and interactions between plants, pathogens and biocontrol agents. The system designed here allowed the isolation of specific factors, such as metabolites and signaling molecules, facilitating a more detailed understanding of the molecular changes and interactions occurring during biocontrol processes. Additionally, the novel hydroponic system can simulate various soil‐less agricultural environments, making the findings more applicable to modern agricultural practices. This method enhances reproducibility and scalability of experiments, providing a robust platform for screening and optimizing biocontrol agents such as *Bv83*.

Based on previous reports,^48^, ^49^, ^50^ in this work we generated a hydroponic system to characterize the molecular dialog between *Bv83*, *A. thaliana* and *B. cinerea*. Our system not only allows us to have full‐grown plants, but also to study the interactions between the biostimulant and the pathogen. Using this hydroponic system, we can not only measure the compounds released either by the plant or by the bacteria into the liquid media, but also study the possible VOCs released by the bacteria that might have an effect over the ISR.

## CONFLICT OF INTEREST

The authors declare that they have no known competing financial interests or personal relationships that could have appeared to influence the work reported in this paper.

## Supporting information


**Figure S1.** VOCs produced by *B. velezensis* 83 are not sufficient to induce ISR against *B. cinerea*. *A. thaliana* roots were incubated with *B. velezensis* 83, 200 mg L^−1^ acetoin or 2,3‐butanediol (applied separately or mixed) and 24 hpt, 5 μL of 5 × 10^4^ spores mL^−1^ of *B. cinerea* were placed over each leaf and incubated for 72 h. The disease incidence was expressed by the percentage of plants showing disease symptoms extending beyond the inoculation site. Histograms represent the mean values (± SE) of three independent experiments. Letters indicate a statistically significant difference, according to one‐way analysis of variance [ANOVA] [*P* ≤ 0.001] followed by a Tukey's HSD test.

## Data Availability

Data sharing not applicable to this article as no datasets were generated or analysed during the current study.
