# Supplementary figures and images for: Bacillus velezensis 83 protects Arabidopsis thaliana against Botrytis cinerea by triggering JA‐, and SA‐dependent induced systemic resistance
Source: Pest Manag Sci. 2025 Nov 19;82(3):2532–40. doi: 10.1002/ps.70390 (PMC12886167; doi:10.1002/ps.70390)

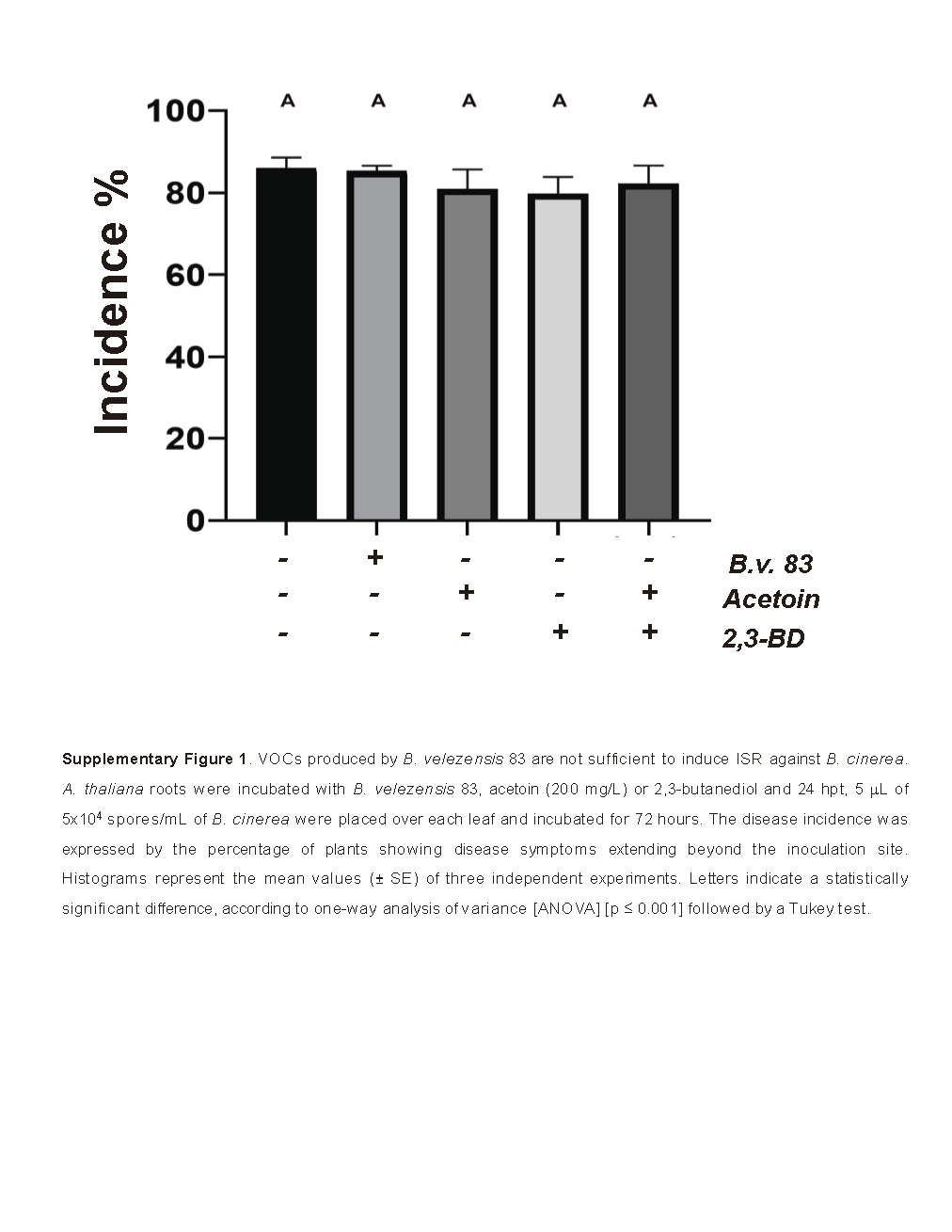

Supplement: Supplementary file 1 — Figure S1. VOCs produced by B. velezensis 83 are not sufficient to induce ISR against B. cinerea. A. thaliana roots were incubated with B. velezensis 83, 200 mg L−1 acetoin or 2,3‐butanediol (applied separately or mixed) and 24 hpt, 5 μL of 5 × 10^4^ spores mL−1 of B. cinerea were placed over each leaf and incubated for 72 h. The disease incidence was expressed by the percentage of plants showing disease symptoms extending beyond the inoculation site. Histograms represent the mean values (± SE) of three independent experiments. Letters indicate a statistically significant difference, according to one‐way analysis of variance [ANOVA] [P ≤ 0.001] followed by a Tukey's HSD test. [file PS-82-2532-s001.tiff]
